# Experimental Investigation of the Melt Shear Viscosity, Specific Volume and Thermal Conductivity of Low-Density Polyethylene/Multi-Walled Carbon Nanotube Composites Using Capillary Flow

**DOI:** 10.3390/polym12061230

**Published:** 2020-05-28

**Authors:** Nicoleta-Violeta Stanciu, Felicia Stan, Catalin Fetecau

**Affiliations:** Center of Excellence Polymer Processing, Dunarea de Jos University of Galati, 47 Domneasca, 800 008 Galati, Romania; Nicoleta.Stanciu@ugal.ro (N.-V.S.); catalin.fetecau@ugal.ro (C.F.)

**Keywords:** melt shear viscosity, shear-thinning, specific volume, transition temperature, thermal conductivity, carbon nanotubes, low-density polyethylene

## Abstract

Understanding the flow behavior of polymer/carbon nanotube composites prior to melt processing is important for optimizing the processing conditions and final product properties. In this study, the melt shear viscosity, specific volume and thermal conductivity of low-density polyethylene (LDPE) filled with multi-walled carbon nanotubes (MWCNTs) were investigated for representative processing conditions using capillary rheometry. The experimental results show a significant increase in the melt shear viscosity of the LDPE/MWCNT composite with nanotube loadings higher than 1 wt.%. Upon increasing shear rates, the composites flow like a power-law fluid, with a shear-thinning index less than 0.4. The specific volume decreases with increasing pressure and nanotube loading, while the *pVT* transition temperature increases linearly with increasing pressure. The thermal conductivity of the LDPE/MWCNT composite is nearly independent of nanotube loading up to the thermal percolation threshold of 1 wt.% and increases linearly with further increases in nanotube loading, reaching 0.35 W/m·K at 5 wt.%. The Carreau–Winter and Cross viscosity models and Tait equation, respectively, are able to predict the shear viscosity and specific volume with a high level of accuracy. These results can be used not only to optimize processing conditions through simulation but also to establish structure–property relationships for the LDPE/MWCNT composites.

## 1. Introduction

Owing to their electrical, thermal, mechanical and chemical properties, complemented by a high surface area [1,2,3,4,5,6,7,8], carbon nanotubes (CNTs) are ideal fillers for a multitude of applications, particularly for structural aerospace, defense and automotive components [9,10,11,12,13,14,15,16,17,18]; conducting components in the field of electronics; sensors and biosensors [2,3,4,5,6,9,10,11,12,13,15,18,19,20,21,22,23,24,25,26,27,28,29,30,31,32]; and coatings and paints [33].

Melt viscosity is one of the most important thermo-physical properties for the polymer processing industry as it is required for product development, tooling design, process optimization, quality control and troubleshooting [34,35,36,37,38]. Specifically, for polymer/CNT composites, melt rheology can be used to assess, in a systematic way, the state of CNTs in polymers (e.g., dispersion, agglomeration and adhesion) after melt processing, which links to the mechanical and electrical properties of the final products [1,3,5,9,10,11,13,15,18,21,22,24,25,39]. Moreover, melt rheology is an important tool for understanding how CNTs affect the processing behavior of polymer/CNT composites [3,5,11,14,15,18,39,40,41].

Although considerable research has been conducted regarding the material properties of polymer/CNT composites, including mechanical, thermal and electrical properties, only a few investigations have focused on the high shear rate rheological behavior [10,11,13,15,18,25,39,40,41,42,43]. Usually, the rheological properties have been measured by parallel plates or con-plates using dynamic oscillation frequency sweeps under linear viscoelastic conditions and shear rates in the range of 0.01 to 100 s^−1^ [1,9,14,19,21,22,24,26,44]. However, in practice, polymer/CNT composites are processed at higher shear rates, such as 10^3^–10^4^ s^−1^ for injection molding [3,5,15,18,36].

Rheological studies on polymer/CNT composites have revealed that the incorporation of CNTs into a polymer matrix brings significant changes in the melt viscosity, which in addition to the temperature and shear rate is affected by the CNT features (type, aspect ratio, loading, and state of dispersion) [1,3,10,11,13,15,18,24,25,39,40,41,42,43]. Specifically, it was reported that the viscosity of polymer/CNT composites usually increases with increasing CNT loading and, at high shear rates, exhibits shear thinning behavior [11,13,15,18,25,40,41,42,43].

The specific volume is also an important thermo-physical property for optimizing the injection molding parameters and predicting the shrinkage or warpage [45,46,47,48] as well as for developing equations of state (EoS) [36,37,45,48,49,50,51,52,53,54]. However, a literature review has shown that there are few polymer/CNT composites for which the pressure–Volume–Temperature (*pVT*) diagrams are available [15,18,41]. Previous studies have emphasized that the specific volume depends on the temperature, pressure and nanotube loadings [15,18,41]. Moreover, for semi-crystalline polymer/CNT composites, the specific volume is also affected by the crystallinity, as CNTs act as nucleating agents for the crystallization [1,16,17,55]. Thus, increasing pressure changes the crystallization conditions, and the transition temperature shifts to higher values, causing different crystalline morphologies [15,18,41,42,51,54].

The thermal conductivity of polymers is another important property for industrial processing like injection molding, in particular for optimizing heat transfer [56]. If the heat transfer is not fully optimized, the process cycle time can be longer than necessary and hot spots can occur, leading to high scrap rates [56]. On the other hand, there are many reasons to benefit from thermally conductive polymer-based composites in various industrial applications, mainly in electronic devices, heat exchangers and functional materials [4,6,7,57]. When conductive fillers are added into polymers, the heat is diffused faster, preventing overheating and thus the premature degradation of the polymer matrix [6]. In particular, among fillers, carbon nanotubes are the most promising candidates due to their high thermal conductivity [28,29,58]. In general, the thermal conductivity of polymer/CNT composites increases with increasing CNT loading, but it is relatively low compared with the thermal conductivity of CNTs [4,7,8,27,30]. The challenge primarily comes from the large interfacial thermal resistance between the CNTs and the polymer matrix, which hinders the motion of phonons through the composite matrix [4,7,27,59]. The orientation of CNTs is also found to have an important influence on the thermal conductivity of polymer/CNT composites [4,6,8,27,60]. In general, it has been shown that moderately oriented polymer/CNT composites exhibit higher thermal conductivity as compared to those randomly oriented [8,27,60].

Computer-aided engineering (CAE) is widely used to simulate melt-based manufacturing processes such as the injection molding process, critical product design aspects (e.g., gate design and location) and defects (e.g., air trap and weld line location and sink marks) can be predicted and corrected, and the processing parameters can be optimized prior to the manufacturing phase when changes are less expensive [36,37,61,62,63]. However, the quality of the simulation results depends on the quality of the material models (i.e., the viscosity models for the fill analysis, thermal properties for the cooling analysis, *pVT* diagrams and mechanical properties for modeling the packing phase and predicting shrinkage and warpage) employed.

An analysis of the polymer databases for simulation tools, such as Moldflow [64] or Moldex3D [65], has shown that the material data (viscosity, *pVT* diagram, thermal conductivity, etc.) for polymer/CNT composites are almost absent. Therefore, it is necessary to create material data that simulation tools/packages do not supply.

In this study, the melt shear viscosity, specific volume and thermal conductivity of low-density polyethylene (LDPE) filled with 0.1–5 wt.% multi-walled carbon nanotube (MWCNT) are investigated using capillary rheometry under experimental conditions similar to those in industrial polymer processing, providing the material parameters and models for numerical simulation.

## 2. Materials and Methods

### 2.1. Materials

The composites investigated in this study were obtained from the PLASTICYL^TM^ LDPE2001 masterbatch (Sambreville, Belgium) that contains 80 wt.% low–density polyethylene (grade ExxonMobil^TM^, Irving, TX, USA, LD 655 [66]) and 20 wt.% of multi–walled carbon nanotubes (grade NC7000^TM^, Nanocyl S.A., Sambreville, Belgium [67]). The LDPE/MWCNT composites with 0.1–5 wt.% of MWCNTs were purchased in the form of pellets from Nanocyl (Sambreville, Belgium). The composites with 1, 3, and 5 wt.% of MWCNTs were obtained by diluting the PLASTICYL^TM^ LDPE2001 masterbatch, whereas the composites with 0.1, 0.3 and 0.5 wt.% of MWCNTs were obtained by diluting the 1, 3 and 5 wt.% compounds, respectively. All the composites were melt-compounded using a twin-screw extruder at 135 °C.

### 2.2. Characterization

#### 2.2.1. Differential Scanning Calorimetry

The crystallization and melting temperatures and the corresponding enthalpies were determined by differential scanning calorimetry (DSC) using a DSC 200 F3 Maia^®^ differential scanning calorimeter (NETZSCH–Gerätebau GmbH, Selb, Germany) at a scanning rate of 10 °C/min in nitrogen (50 mL/min). The instrument was calibrated with indium at various heating rates, and the baseline was obtained by scanning the temperature domain with an empty pan. To erase the thermal history, first, the samples with an average mass of ~20 mg were heated from 20 °C to 200 °C at a heating rate of 10 °C/min. The samples were then cooled down to room temperature at 10 °C/min and heated again at the same rate to 200 °C.

The crystallinity of the LDPE/MWCNT composites was calculated with

(1) $$\chi =\frac{\Delta H_{m}}{\Delta H_{100}(1-\varphi )}\cdot 100\%,$$

where $\Delta H_{m}$ is the melting enthalpy of the sample, $\Delta H_{100}$ is the melting enthalpy for 100% crystalline polyethylene (293 J/g [9]), and φ is the mass weight fraction of the MWCNTs.

#### 2.2.2. Melt Shear Viscosity

The rheological properties were measured using a Rheograph 75 capillary rheometer (Göttfert GmbH, Buchen, Germany) at shear rates ranging from 50 to 5000 s^−1^ and temperatures spanning the processing temperature window (110 to 150 °C). The tests were performed on three capillary dies with length-to-diameter ratios—*L* (mm)/*D* (mm)—of 10/1, 20/1 and 30/1, respectively, and the Bagley (i.e., pressure loss) and Weißenberg–Rabinowitsch (i.e., pseudo-plastic behavior) corrections were applied using the WinRheo II software (Göttfert, Germany) [68].

Prior to each experiment, the composite pellets were dried in a vacuum oven at 80 °C for 2 h (Raypa, Terrassa, Spain). To ensure that the LDPE/MWCNT composites experienced the same thermo-mechanical history, the pellets were loaded into the barrel at the test temperature and melted for 300 s.

In order to investigate the behavior of the LDPE/MWCNT composites under shear rates, thermo-stability tests were performed using the die with the *L/D* ratio of 20/1. After the LDPE/MWCNT pellets were melted for 600 s into the preheated barrel at 130 °C, a constant shear rate of 200 s^−1^ was applied and the pressure was recorded as a function of time.

#### 2.2.3. Specific Volume

The pressure–Volume–Temperature (*pVT*) measurements were carried out according to ISO 17744 using the Rheograph 75 (Göttfert GmbH, Buchen, Germany) equipped with a *pVT* piston with a Teflon ring and a capillary quick-lock system. The dried pellets were gradually loaded into the barrel heated at 110–115 °C (depending on the nanotube loading) and compressed with a force of 15 kN to ensure that no air bubbles are trapped inside the melt. After fully loading the barrel, the *pVT* piston was inserted until 0.5 kN force was applied on the melt and held for 5 min to accommodate the Teflon ring. Next, the melt was extruded until about 18 mm of sample was left in the barrel and heated with an average heating rate of 5 °C /min to a temperature of 160 °C to ensure the full melting of the crystalline structure. To capture the crystallization region, the specific volume was measured during cooling using an isothermal mode in order of decreasing temperature from 160 °C to 30 °C, at 5 °C intervals. For each temperature, the melt was pressurized to the desired pressure level (in increasing order from 10 to 1500 bar). Thus, the sample reached the lowest temperature only once, at the end of the cycle. The relaxation time between two successive temperatures was set to 30 min.

#### 2.2.4. Thermal Conductivity

The thermal conductivity (TC) was measured according to ASTM D5930 using the same capillary rheometer (Göttfert GmbH, Buchen, Germany), which was equipped with a quick-lock system and a thermal conductivity probe. The TC probe consists of a thin-walled piston with a thermocouple and a heating element in the center. The LDPE/MWCNT pellets were loaded into the preheated barrel at 110–115 °C (depending on the nanotube loading) and compressed using a standard test piston until a 15 kN force was reached. After the barrel was filled in, the standard test piston was removed and the TC probe was inserted into the composite melt and extruded until about 50 mm of sample was left in the barrel. The TC was measured during cooling mode in order of decreasing temperature from 140 to 70 °C, at 10 °C intervals, whereas the pressure was scanned in increasing order (100, 200, 250 and 500 bar). A dwell time of 15 min was set before starting a measurement to achieve the thermal equilibrium. For each applied pressure, two consecutive measurements were taken to check the reproducibility. The TC was calculated from the temperature increase and the heat flow (i.e., from the slope of the heat flow generated at 69 volts vs. temperature).

#### 2.2.5. Solid Density

The solid density measurements were carried out according to ISO 1183–1 using a Density Kit for the Mettler Toledo analytical balance (AB204–S/FACT, Mettler Toledo Inc., Columbus, OH, USA). The principle is described in [18]. All the measurements were carried out at room temperature, around 23 °C, and the reported results are the averages of ten replicate measurements.

## 3. Results and Discussion

### 3.1. Thermal Properties

As different studies have shown, carbon nanotubes act as nucleating agents, influencing the crystallization process of semi-crystalline polymers [1,2,9,12,16,17,20,23,55,69]. The crystallization process is characterized first by crystal nucleation and second by crystal growth, and is accelerated due to the presence of carbon nanotubes as a result of the heterogeneous nucleation process [2,17,20,55,69]. However, carbon nanotube networks can obstruct/restrict the diffusion of polymer chains, resulting in a slower crystallization rate [2,20,69], and/or block the crystal growth due to the formation of an external barrier (i.e., the barrier effect of carbon nanotubes). On the other hand, CNTs can be encapsulated in the LDPE matrix due to its crystallization, leading to little or no interaction between the nanotubes and the polymer [9,17,23].

**Figure 1:**
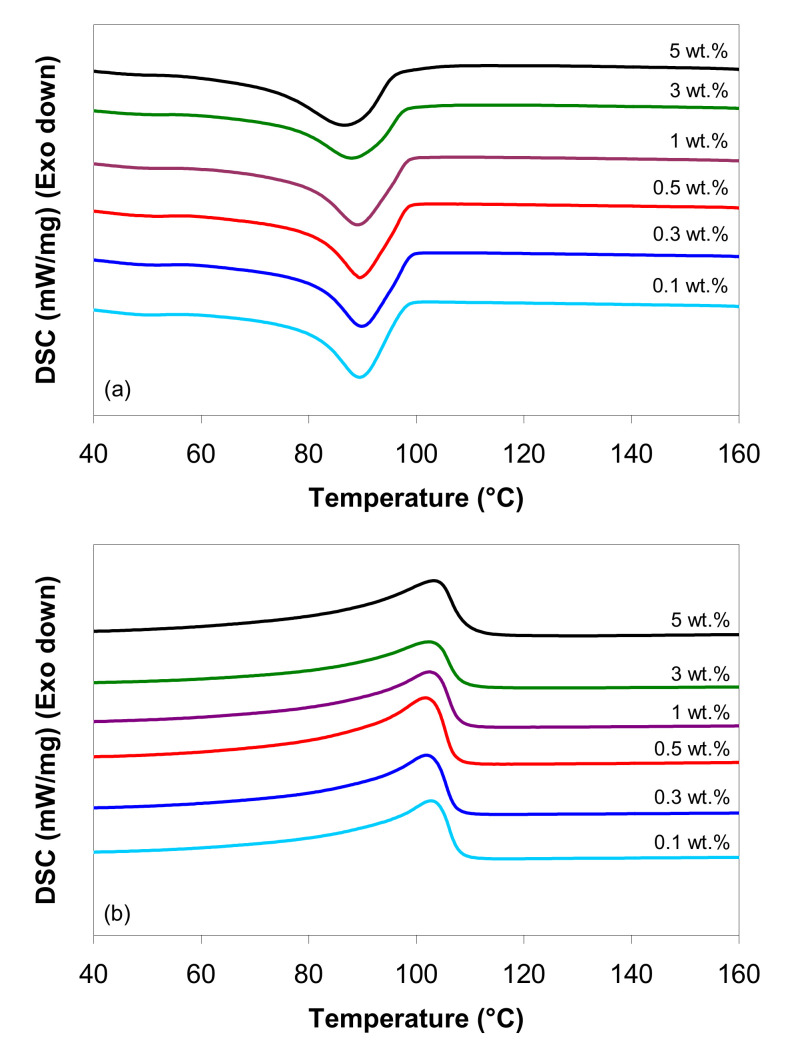
Differential scanning calorimetry (DSC) curves of low-density polyethylene (LDPE)/ multi-walled carbon nanotube (MWCNT) composites: (**a**) first cooling and (**b**) second heating at a 10 °C/min rate (the DSC scans are offset for clarity).

Figure 1 shows the DSC cooling scan and the subsequent heating scan performed at 10 °C/min, and Table 1 summarizes the thermal properties (e.g., $T_{m}$—melting temperature, $\Delta H_{m}$—melting enthalpy, $T_{c}$—crystallization peak temperature, $\Delta H_{c}$—crystallization enthalpy, $T_{s}$—ejection temperature, and *χ*—crystallinity). It can be seen that the DSC curves for the LDPE/MWCNT composites are not significantly different, and the nucleation activity associated with the nanotubes increases weakly with nanotube loading. These results are broadly in line with other research [9,10,23].

The DSC exothermic curves in Figure 1a show that the LDPE/MWCNT composites have a $T_{c}$ that is nearly constant at around ~90 °C for the nanotube loading up to 1 wt.%. At higher nanotube loadings, the $T_{c}$ is shifted towards lower values. These changes are caused by the variations in the nucleation activity of the carbon nanotubes. When a small amount of carbon nanotubes is incorporated (0.1–0.5 wt.%), the nucleation density increases significantly. When the nanotube loading is higher than 1 wt.%, the crystallization process is delayed; due to the MWCNT aggregation and/or the barrier effect of carbon nanotubes, the polymer chains undergo restriction of movement, postponing the crystal growth and the formation of new interfacial regions [12,70]. As can be seen in Table 1, the enthalpy of crystallization varies randomly between 68 and 92 J/g, suggesting differences in the homogeneity of the dispersion in the LDPE matrix.

The results from the first DSC heating scan (Table 1) show that the addition of CNTs increased the melting temperature by about 3–6 °C, as compared to the melting peak temperature of the LDPE matrix (101 °C); however, the effect of nanotube loading is not consistent as no trend regarding the dependence on the MWCNT loading was observed.

The results from the second DSC heating scan indicate that the melting peak temperature of the LDPE/MWCNT composites, located at around 103 °C (Figure 1b and Table 1), was not affected by the presence of nanotubes, i.e., the melting peak temperature varied by ~1 °C, which is within experimental error. However, for the second heating (Figure 1b), the peak melting temperature was shifted about 2–3 °C towards lower temperatures, as compared with the first heating scan. The DSC endothermic curves in Figure 1b indicate a melting point that is located at around 103 °C.

The LDPE/MWCNT composites show a significant change in the enthalpy of melting values from 60 to 90 J/g for MWCNT loading, ranging from 0.1 to 5 wt.% (Table 1), but without a clear trend. The results of the second heating scan show that the % crystallinity of the LDPE/MWCNT composites has an increasing tendency compared with the first heating scan. The highest % crystallinity was obtained for the 5 wt.% of MWCNT composite, while the lowest % crystallinity was observed for the composite with 3 wt.%.

### 3.2. Thermo-Stability of LDPE/MWCNT Composites

**Figure 2:**
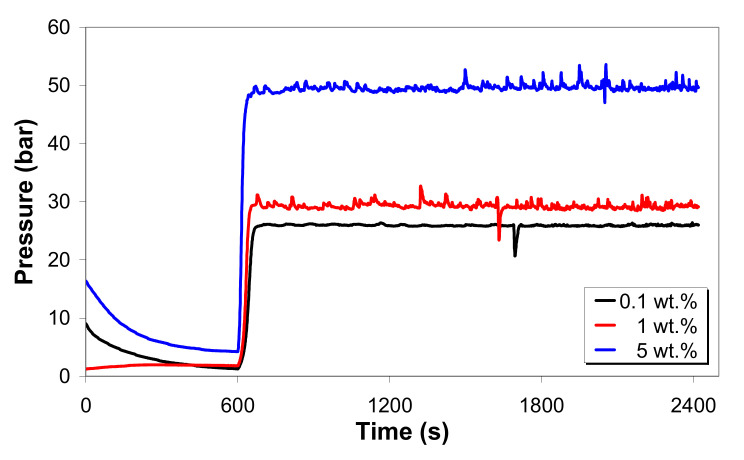
Pressure versus time at a 200 s^−1^ apparent shear rate and 130 °C melt temperature (capillary die ratio, L/D = 20).

Figure 2 shows the thermo-stability curves for the LDPE/MWCNT composite with 0.1, 1 and 5 wt.% of MWCNTs. Under a constant shear rate of 200 s^−1^, the composite with 5 wt.% of MWCNTs withstands higher pressure (~50 bar) compared to the composites with 1 wt.% (~30 bar) and 0.1 wt.% (~25 bar). The composite melts do not undergo any significant shear degradation at different carbon nanotube loadings as no discontinuities in the flow process or pressure loss were observed. However, the LDPE/MWCNT composites experienced some melt flow instability (i.e., the weak tearing and stick–slip of polymer chains) as indicated by the pressure fluctuation in Figure 2.

It is also seen that the pressure oscillations increased with increasing nanotube loading. In part, this could be due to the tearing and slipping of the polymer chains attached to the wall and sudden disentanglement of the polymer chains and CNT network [71]. However, the pressure fluctuation is less than 2 bar, and the extrudates that come out of the die are continuous with a smooth surface. It should be noted that the lowest peaks may be associated with the presence of air bubbles.

### 3.3. Effect of Pressure on Capillary Flow

The pressure drop over the capillary length as a function of the *L*/*D* ratio for a given shear rate (so-called Bagley plots) can give useful information on the (constant shear rate) pressure dependence of viscosity [52]. The linearity of the Bagley plots indicates that the effect of viscous heating on the viscosity is not significant, whereas deviations from linearity are usually attributed to the pressure dependence of viscosity or to orientation effects [52,72].

**Figure 3:**
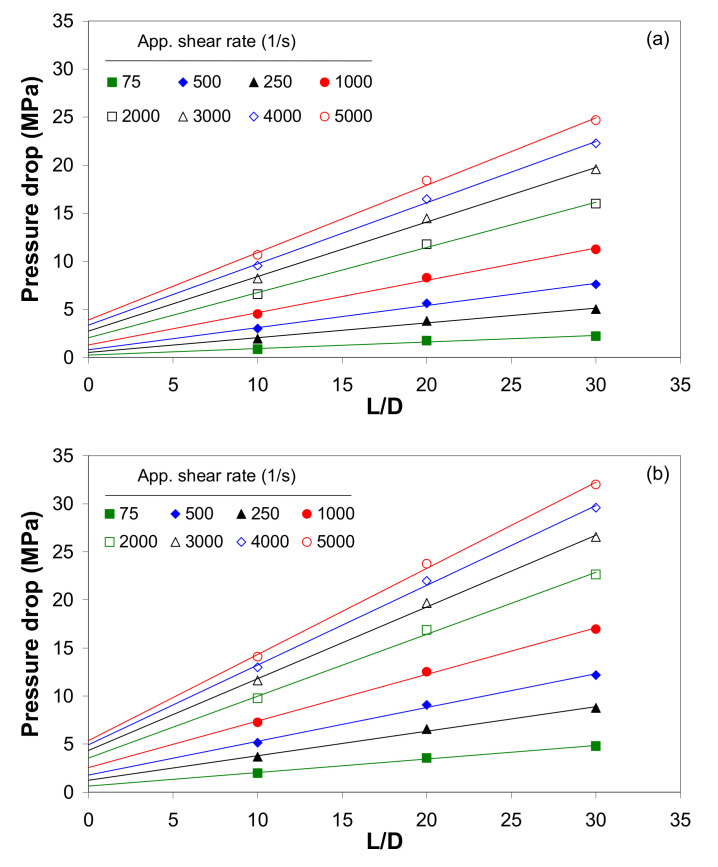
Bagley plot for the LDPE/MWCNT composite with (**a**) 0.1 wt.% and (**b**) 5 wt.% of MWCNTs at 120 °C.

Figure 3 shows the Bagley plots for the composites with 0.1 and 5 wt.% of MWCNTs at 120 °C for apparent shear rates ranging from 50 to 5000 s^−1^. The pressure drop increases with increasing MWCNT wt.%, and the increase in pressure drop is more evident at higher shear rates. For example, at a shear rate of 5000 s^−1^, the pressure drop increased from about 3.9 MPa to 5.4 MPa as the nanotube loading increased from 0.1 to 5 wt.% of MWCNTs. The determination coefficients (*R*^2^) of the regression lines were used to assess the linearity of the Bagley plots. The analysis of these plots shows that the data in the Bagley plots fall on a straight line (with *R*^2^ > 0.994), even at low shear rates, indicating that with short durations, the pressure effect is not significant. By investigating the pressure drop across the capillary from different sets of results, it could be concluded that the short-term effect of pressure on the shear viscosity is not significant.

### 3.4. Apparent Shear Stress

**Figure 4:**
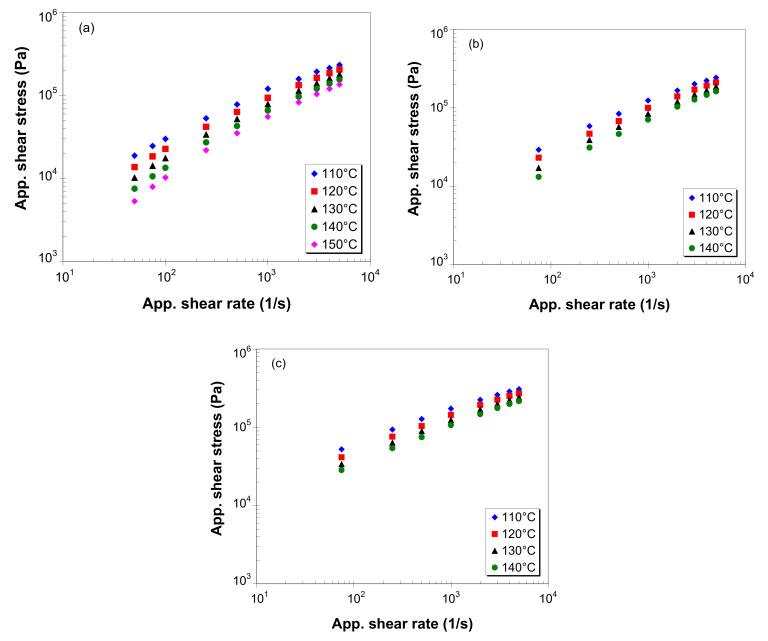
Apparent shear stress vs. apparent shear rate for LDPE/MWCNT composites with (**a**) 0.1 wt.%, (**b**) 1 wt.% and (**c**) 5 wt.% of MWCNTs (*L/D* = 30).

Figure 4 shows the flow curves, i.e., the apparent shear stress versus apparent shear rate, of LDPE/MWCNT composites for different temperatures and MWCNT loadings, on a double logarithmic scale. Overall, the apparent shear stress increases with an increasing apparent shear rate and decreases with an increasing melt temperature. Moreover, the shear stress increases with increasing MWCNT loading.

At low nanotube loadings (0.5 wt.% and below) and low shear rates (100 s^−1^ and below), as shown in Figure 4a, the logarithm of the apparent shear stress deviates from a linear shape, indicating that the LDPE/MWCNT composite exhibits Newtonian flow behavior. However, at higher nanotube loadings, as shown in Figure 4c, the LDPE/MWCNT composite exhibits non-Newtonian behavior even at low shear rates (50–100 s^−1^).

To describe the apparent shear stress, τ, as a function of the shear rate, $\dot{\gamma }$, the power-law model was considered [35,73]:

(2) $$\tau =K\cdot {\dot{\gamma }}^{n}$$

where K is the consistency index and n is the power-law index.

The best-fit parameters are given in Supplementary Table S1, for each nanotube loading and temperature. It can be observed that the K index decreases nonlinearly with increasing melt temperature (the variation is consistent with the power-law model) and dramatically increases with increasing nanotube loading. The n index, on the other side, increases linearly with increasing melt temperature and decreases with increasing nanotube loading. As shown in Supplementary Table S1, the $R^{2}$ value increases with increasing MWCNT loading, indicating an increase in linearity and, in particular, an increase in shear thinning—as the nanotube loading increases, nanotube–nanotube interactions begin to dominate and lead to shear thinning.

The analysis of variance presented in Supplementary Tables S2 and S3 shows that both melt temperature and nanotube loading have a significant effect on the parameters *K* and *n*, and the effect of nanotube loading is more important (~65%) than the effect of temperature (~30%).

### 3.5. Effect of Temperature on Melt Shear Viscosity

**Figure 5:**
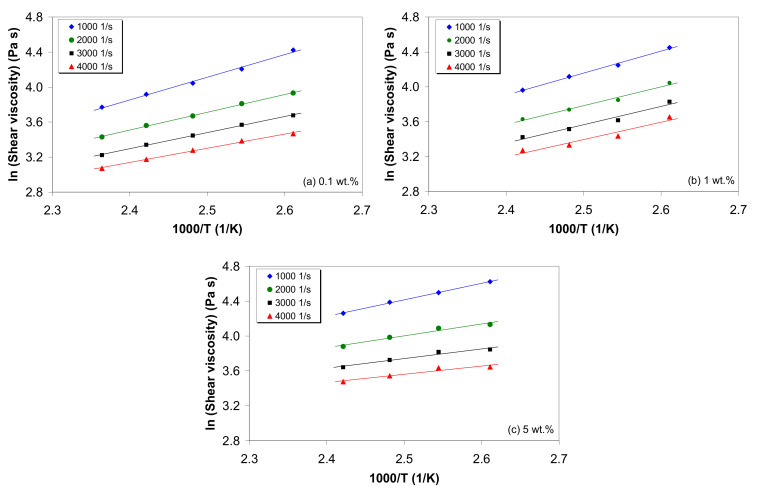
Arrhenius plots for the melt shear viscosity of LDPE/MWCNT composites with (**a**) 0.1 wt.%, (**b**) 1 wt.% and (**c**) 5 wt.% of MWCNTs at different shear rates.

The effect of temperature on the melt shear viscosity of LDPE/MWCNT composites is illustrated in Figure 5, for selected shear rates and nanotube loadings. At a constant shear rate and nanotube loading, the effect of temperature on melt shear viscosity is well represented by an Arrhenius-type equation [35,73]:

(3) $$\eta =A\exp\left(\frac{E_{\dot{\gamma }}}{R_{g}}\frac{1}{T}\right),$$

where η is the melt shear viscosity, A is the viscosity constant, $E_{\dot{\gamma }}$ is the activation energy for flow, T is the absolute temperature and $R_{g}$ is the molar gas constant ($R_{g}=8.31446$ J·K^−1^·mol^−1^).

The flow activation energy, determined from the slope ($E_{\dot{\gamma }}/R_{g}$) of the lines in Figure 5, and the determination coefficient ($R^{2}$) are reported in Supplementary Table S4. It can be seen that the linearity holds very well for all shear rates and MWCNT loadings, as indicated by the $R^{2}$ values.

**Figure 6:**
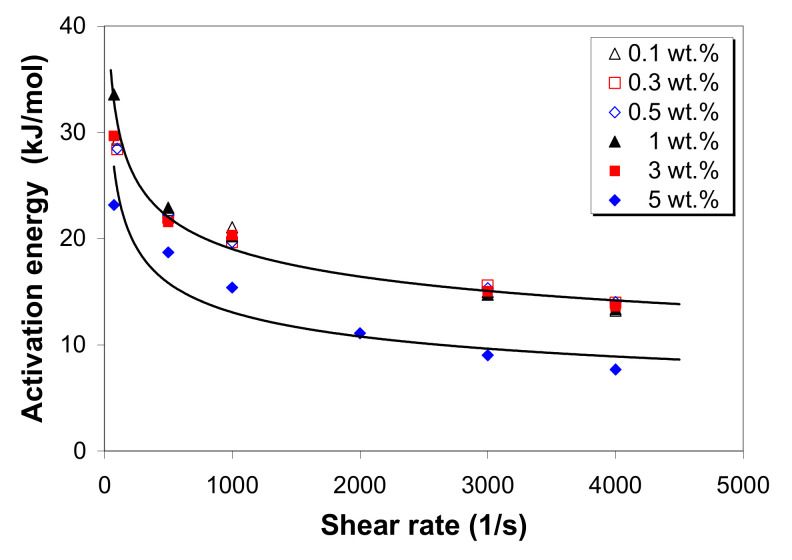
Effects of the shear rate and MWCNTs on the flow activation energy of LDPE/MWCNT composites.

Figure 6 shows the influence of the shear rate and MWCNT loading on the flow activation energy of the LDPE/MWCNT composites. It can be seen that the activation energy decreases nonlinearly with increasing shear rate, suggesting that the sensitivity of the viscosity to temperature reduces with the shear rate. Generally, the higher the activation energy, the more temperature-sensitive the melt is [10,18,22,40,43]. In addition to the shear rate, carbon nanotube loading has a significant influence on the activation energy. The activation energy was found to only slightly decrease with increasing nanotube loading up to 3 wt.%, after which it decreases sharply with a further increase in nanotube loading. The decrease in the activation energy with increasing MWCNT loading can also be explained by the increase in the CNT–CNT interactions as a result of less interaction with the polymer chains [10,22]. This is an indication that the sensitivity of the melt shear viscosity to temperature variation reduces with increasing nanotube loading. This is in good agreement with the sensitivity of viscosity characterized by the shear thinning. The 5 wt.% composite exhibits the lowest power-law index and, therefore, more shear thinning and accordingly less resistance to flow.

The ANOVA for the flow activation energy (Supplementary Table S5) shows that the shear rate has the most important contribution to the activation energy (~78%), followed by the nanotube loading with a contribution of about 21%, and these effects are statistically significant at all levels.

### 3.6. Effect of MWCNTs on Melt Shear Viscosity

**Figure 7:**
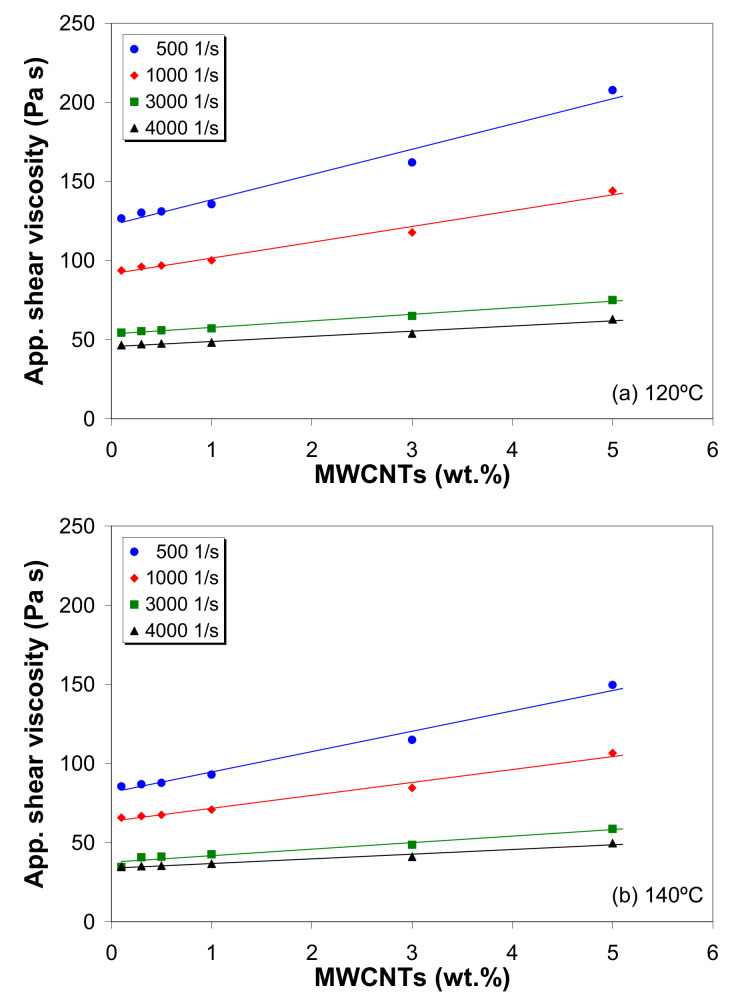
Relationship between apparent shear viscosity and MWCNT loading at (**a**) 120 °C and (**b**) 140 °C.

The viscosity of polymer/CNT composites is generally influenced by the carbon nanotube loading through nanotube–nanotube and nanotube–polymer matrix interactions [10,22,39,42,43]. Figure 7 shows the dependence of the apparent shear viscosity on the MWCNT loading, at 120 °C and 140 °C and with shear rates ranging from 500 to 4000 s^−1^. A linear dependence of the apparent shear viscosity on nanotube loading can be observed in Figure 7, as indicated by the solid lines. The corresponding fitting parameters are given in Supplementary Table S6.

With respect to the changes in apparent shear viscosity, a linear increase in melt shear viscosity with increasing MWCNT loading is observed, but the sensitivity of the shear viscosity to the nanotube loading is weakened with increasing apparent shear rate and temperature. At low shear rates, the movements of the macromolecule chains of the LDPE melt are blocked by the presence of carbon nanotubes, which eventually form CNT–CNT networks. This leads to an increase in flow resistance during capillary extrusion, which increases with increasing MWCNTs wt.%. On the other hand, when the melt is subjected to high shear rates, the nanotubes align along the shearing direction, which causes a decrease in melt flow resistance through capillaries and, therefore, a decrease in viscosity. These findings are consistent with [10,18,40,41,42,43].

### 3.7. Modeling of Melt Shear Viscosity

The power-law model (as in Equation (2)), in general, is used to describe the flow behavior in the range of medium to high shear rates. However, during melt processing, polymer melts are subjected to both low and high shear rates [36,37]. Therefore, the flow model must be able to describe the viscosity at both low and high shear rates [34,35,73]. Moreover, in most CAE programs for simulating polymer flow, the melt shear viscosity is a single function of both the shear rate and temperature. Therefore, based on the corrected melt shear viscosity, master curves were constructed using the WinRheo II software [68] by employing the time–temperature superposition principle at a reference temperature of 130 °C. Then, the experimental master curves were fitted to the Carreau–Winter [34,68,73] and Cross [35,68,73] viscosity models, respectively:

(4) $$\eta =\frac{\eta_{0}}{{\left(1+\lambda \dot{\gamma }\right)}^{m_{c}}},$$

(5) $$\eta =\frac{\eta_{0}}{1+{\left(\frac{\eta_{0}}{\tau *}\dot{\gamma }\right)}^{1-n}},$$

where $\eta_{0}$ is the zero-shear viscosity, corresponding to the lower Newtonian plateau; λ is the transition time; $m_{c}$ is the viscosity exponent; τ* is the shear stress at which the transition to non-Newtonian behavior begins; and n is the power-law exponent.

**Figure 8:**
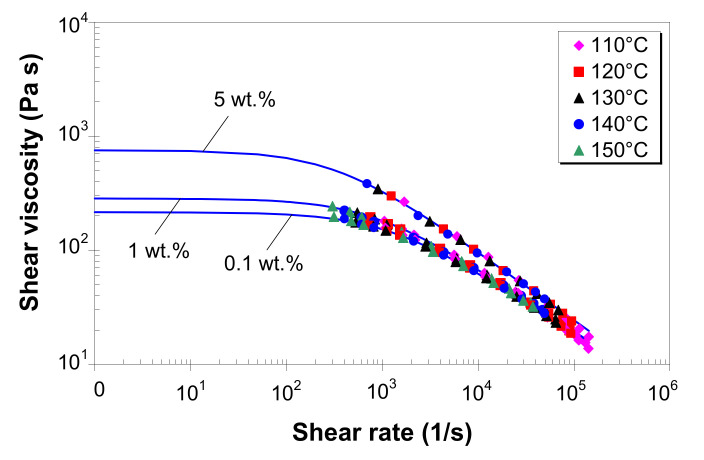
Viscosity master curves at a reference temperature of 130 °C, and predictions (solid lines) by the Carreau–Winter model.

Figure 8 shows the experimental viscosity master curves for LDPE/MWCNT composites with 0.1, 1 and 5 wt.% of MWCNTs. Solid lines represent the predictions from the Carreau–Winter model, with the coefficients listed in Table 2. The Carreau–Winter model describes very well the experimental data for all the composites in the entire experimental shear rate range, as demonstrated by the $R^{2}$ values in Table 2. In addition, at very low shear rates, the Carreau–Winter model predicts a Newtonian plateau, which increases with increasing MWCNT loading.

**Figure 9:**
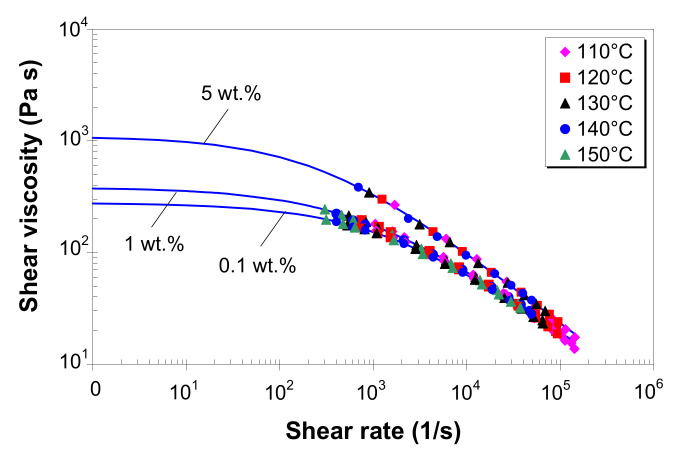
Viscosity master curves at a reference temperature of 130 °C, and predictions (solid lines) by the Cross model.

Figure 9 shows the master curves for the LDPE/MWCNT composite with 0.1, 1 and 5 wt.% of MWCNTs as predicted by the Cross model (solid lines), with the coefficients listed in Table 3. As shown in Figure 9, the Cross model perfectly fits the experimental master curves ($R^{2}$ values close to 1, in Table 3). At low shear rates, for nanotube loading up to 3 wt.%, the Cross model predicts a Newtonian plateau, whereas for 5 wt.%, the Cross model predicts a much steeper slope.

All the LDPE/MWCNT composites exhibited solid-like behavior, and the shear-thinning exponent *n* decreased with increasing MWCNT loading (Table 3). This behavior is an indication that interactions between MWCNT and MWCNT are dominant as the MWCNT loading increases, and the shear rate affects both the nanotube network and the polymer entangled network.

**Figure 10:**
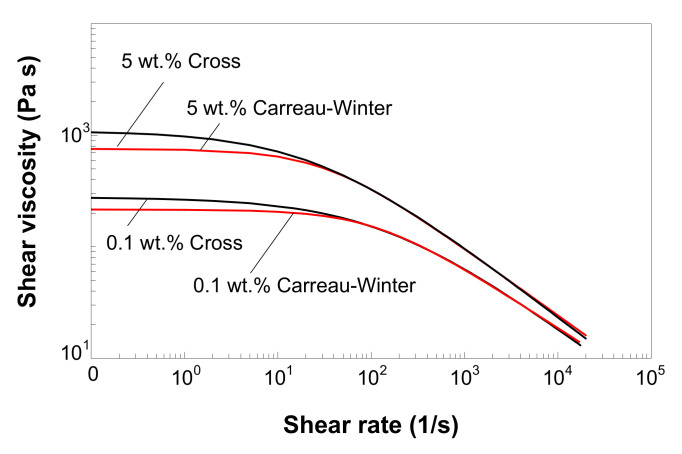
Comparison between the melt shear viscosity predictions by the Cross and Carreau–Winter models (master curves at 130 °C reference temperature).

The direct comparison of the two viscosity models in Figure 10 shows that the viscosity from the Carreau–Winter model compares very well with that from the Cross model, except for (i) shear rates lower than 100 s^−1^, where the Carreau–Winter model asymptotes to lower $\eta_{0}$ values than the Cross model, and (ii) shear rates higher than 10^4^ s^−1^, where the Carreau–Winter model slightly over-predicts the viscosity.

The Carreau–Winter model predicts lower $\eta_{0}$ values (Table 2) as compared with the Cross model (Table 3) because the Carreau–Winter model has only one adjustable parameter, i.e., $m_{c}$, thus it is able to describe more gradually the transition from Newtonian plateau to the shear-thinning region. On this point, it is important to highlight the fact that the applicability of the viscosity model to predict the $\eta_{0}$ viscosity is limited due to the absence of experimental data at apparent shear rates lower than 50 s^−1^. Consequently, given the empirical nature of these predictions, the $\eta_{0}$ viscosity cannot represent exactly the rheological response of LDPE/MWCNT composites at low shear rates. Moreover, its relevance for practical applications is limited by the fact that the zero-shear viscosity virtually never occurs in melt processes.

### 3.8. Pressure–Volume–Temperature Behavior of LDPE/MWCNT Composites

**Figure 11:**
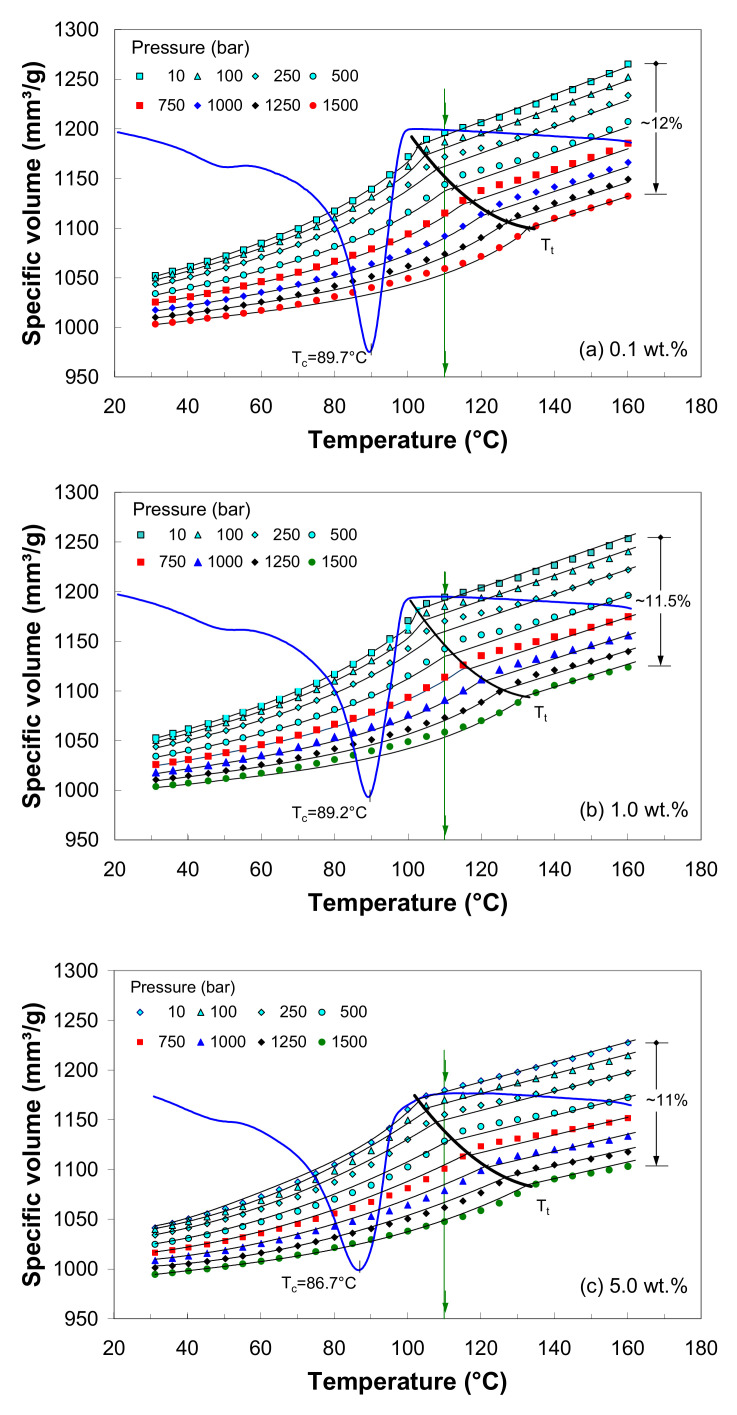
*pVT* diagram of the LDPE/MWCNT composite with (**a**) 0.1 wt.%, (**b**) 1 wt.% and (**c**) 5 wt.% of MWCNTs. The solid lines show data fitting to the Tait model.

Figure 11a–c show the *pVT* data for LDPE/MWCNT composites with 0.1, 1 and 5 wt.% of MWCNTs, respectively. As mentioned in the experimental section, the *pVT* tests were performed in (isobaric) cooling mode. Therefore, to enable comparison, additionally, the corresponding DSC cooling curves are shown in Figure 11.

The *pVT* diagrams in Figure 11 display three distinct regions corresponding to the melting, transition and semi-crystalline states, respectively. Generally, at $T>T_{m}$, the composites must be treated as a mixture of LDPE melt and MWCNTs. At $T_{c}\leq T\leq T_{m}$, a mixture of crystals, MWCNTs and polymer melt exists, while at $T<T_{c}$, the composite can be regarded as a semi-crystalline solid.

The decrease in the specific volume is associated with the crystallization process. Therefore, the solid curve $T_{t}=T(p)$ in Figure 11 indicates the onset of the crystallization process (i.e., below this curve, the melt starts to crystallize), which is clearly dependent on the pressure. At 110 °C and 10 bar pressure, the composite is well above the melting temperature and is highly expanded. As the pressure increases above 500 bar, the composite melt starts to crystallize, as can be seen by following down the line at 110 °C (Figure 11), and the crystallization process shifts towards higher temperatures with increasing pressure (e.g., if the composite melt is subjected to 1500 bar, the onset crystallization occurs at about 140 °C).

From Figure 11, it is evident that the LDPE/MWCNT composites crystallize at temperatures higher than the DSC crystallization temperature through increasing the applied pressure. However, at low pressure (10 bar or below), the DSC onset crystallization temperature is in good agreement with the *pVT* onset transition temperature.

Figure 11 also indicates that the specific volume decreases with increasing pressure. With increasing pressure, the specific volume change gets smaller and smaller, which corresponds to a decrease in compressibility. In the solid state, the change in the specific volume (the solid compressibility) is much smaller than that in the melt state (the melt compressibility).

Overall, the change in specific volume within the *p-T* window is about 5% to 15%, depending on the nanotube loading. When the effect of MWCNT loading is separated from that of melt temperature and pressure, the results suggest that the effect of the temperature is apparently more significant than the effect of the pressure due to the phase changes that occur in the investigated temperature range. The decrease in the specific volume with regard to the applied pressure is the same for every nanotube loading, about 11%–12% and 5% in the melt and solid state, respectively.

Specifically, in the case of 0.1 wt.% of MWCNTs (Figure 11a), the specific volume of the LDPE/MWCNT composite is 1052.10 mm^3^/g at 30 °C and 10 bar, roughly 5% higher than that at 1500 bar. In the melt state, e.g., at 160 °C, the composite with 0.1 wt.% has a specific volume of 1265.26 mm^3^/g at 10 bar, while at 1500 bar, the specific volume is 1132.12 mm^3^/g; this is equivalent to a decrease in the specific volume of 12%. Furthermore, the crystallization starts at about 103 °C at 10 bar, which is in agreement with the DSC data (Table 1), whereas the onset of the crystallization process is shifted about 30 °C higher at 1500 bar, 30 °C higher than that at 10 bar. Based on the experimental data, the following relation was obtained for the onset transition temperature: $T_{t}(p)/({}^{\circ}C)=102.51+0.0204\cdot p/(bar)$.

For the LDPE/MWCNT composite with 1 wt.%, at 10 bar, the specific volume varies from 1052.95 mm^3^/g at 30 °C to 1253.71 mm^3^/g at 160 °C, an increase of 19%. At 1500 bar, the specific volume variation is only 12% and is 1004.14 mm^3^/g and 1124.30 mm^3^/g at 30 °C and 160 °C, respectively. As can be seen in Figure 11b, the crystallization starts at about 103 °C at 10 bar, and the pressure dependence of the transition temperature may be approximated by $T_{t}(p)/({}^{\circ}C)=102.51+0.0220\cdot p/(bar)$.

At 10 bar, the specific volume of the LDPE/MWCNT composite with 5 wt.% of MWCNTs (Figure 11c) varies from 1041.23 mm^3^/g at 30°C to 1227.55 mm^3^/g at 160°C, an increase of 18%. At 1500 bar, the specific volume variation is only 11% and ranges from 994.39 mm^3^/g at 30°C to 1103.09 mm^3^/g at 160°C. Similarly, at 10 bar, the onset of crystallization takes place at about 103°C. For the composite with 5 wt.% of MWCNTs, the pressure dependence of the transition temperature may be approximated by $T_{t}(p)/({}^{\circ}C)=102.72+0.0192\cdot p/(bar)$.

The dependence of the crystallization temperature on the pressure is particularly important for melt-manufacturing processes, which require cooling under pressure. For example, during the packing stage of the injection molding process, the melt is held constant under high pressure [45,46,47,48], which may induce changes in crystallization and, consequently, in the final properties of the parts. This effect is even more important in the presence of nanotubes, which provide sites for the nucleation process [2,16,17,20,55]. As illustrated in Figure 11, the crystallization temperature of the LDPE/MWCNT composites may be increased by about 25 °C when the composite is cooled under pressure, which may lead to better mechanical performance when subjected to various environmental stresses [47,48,50,54]. This finding depicts a potential way to modify the morphology of the manufactured composites.

**Figure 12:**
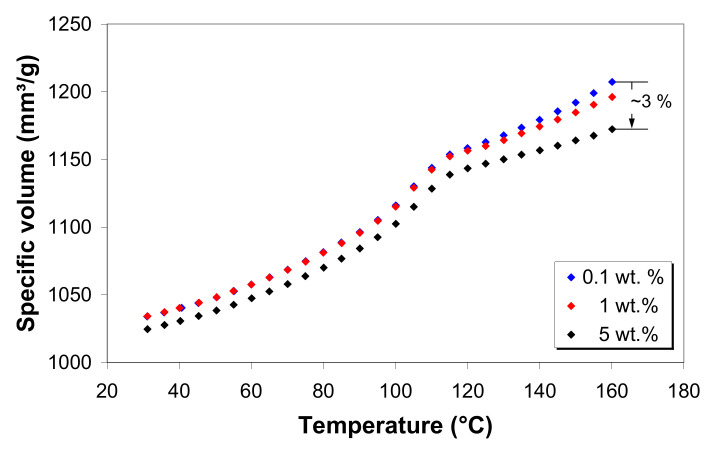
Effect of melt temperature and nanotube loading on the specific volume of the LDPE/MWCNT composites at 500 bar.

To illustrate the effect of MWCNT loading on the specific volume, the data for 0.1, 1 and 5 wt.% of MWCNT are compared in Figure 12 at a pressure of 500 bar. It can be seen that, in the solid state, the specific volume is nearly constant with increasing nanotube loading up to 1 wt.%, where a further increase results in a decrease in the specific volume. However, in the melt state, the specific volume shows a general decrease with nanotube loading, and the effect is more important at higher nanotube loading.

### 3.9. Modeling of the Specific Volume of LDPE/MWCNT Composites

The specific volume, necessary to account for the compressibility of polymers in melt processing such as injection molding and extrusion, is generally described by the Tait equation [36,37,45,46]. Although this equation is purely empirical, the Tait model is widely used to model the *pVT* behavior of both semi-crystalline and amorphous polymers [49,50,51,52,53,54]. However, the Tait equation best describes the behavior of the amorphous polymers because they do not exhibit any crystalline structure and, from room temperature to softening point, only one transition occurs (e.g., the glass transition temperature) [50,52,53], while for crystalline polymers, due to the pressure- and temperature-dependence of the crystallization, the evaluation of the Tait equation is slightly more complicated [49,51,54,74].

According to the Tait model, the specific volume as a function of pressure and temperature is given by [36,45]:

(6) $$V\left(T,p\right)=V_{0}\left(T\right)\cdot \left[1-C\cdot \ln\left(1+\frac{p}{B\left(T\right)}\right)\right]+V_{t}\left(T,p\right),$$

in which $V_{0}(T)$ is the specific volume at atmospheric pressure,

(7) $$V_{0}(T)=\left\{\begin{array}{ll} b_{1s}+b_{2s}\cdot \tilde{T} & T\leq T_{t}\\ b_{1m}+b_{2m}\cdot \tilde{T} & T>T_{t} \end{array}\right.,$$

$V_{t}(T,p)$ is the specific volume decrease due to the crystallization process,

(8) $$V_{t}\left(T,p\right)=\left\{\begin{array}{ll} b_{7}\cdot \exp\left(b_{8}\cdot \tilde{T}-b_{9}\cdot p\right) & T\leq T_{t}\\ 0 & T>T_{t} \end{array}\right.,$$

and B(T) is the pressure sensitivity,

(9) $$B(T)=\left\{\begin{array}{ll} b_{3s}\cdot \exp\left(-b_{4s}\cdot \tilde{T}\right) & T\leq T_{t}\\ b_{3m}\cdot \exp\left(-b_{4m}\cdot \tilde{T}\right) & T>T_{t} \end{array}\right.,$$

where $\tilde{T}=T-b_{5}$. The constant C in Equation (6) has a typical value of 0.0894 [36,45].

The transition temperature is generally assumed to be a linear function of pressure [36,45]:

(10) $$T_{t}(p)=b_{5}+b_{6}\cdot p.$$

For amorphous polymers, the transition temperature represents the glass transition, while for semi-crystalline polymers, the transition temperature is the melting or crystallization temperature, depending on whether the *pVT* test is conducted in heating or cooling mode.

In Equations (7)–(9), the s subscript refers to solid state, while the m subscript refers to melt state. Parameter $b_{1m}$ is the specific volume at $b_{5}$ (the intercept of the melt-state V−T line at p=0). Parameter $b_{2m}$ represents the slope of the V−T line at p=0 in the melt state. Parameter $b_{3m}$ is the pressure sensitivity above $T_{t}$ (line drop) or the spread of the melt, while $b_{4m}$ is the pressure sensitivity of the melt slope. The coefficients $b_{5}$ and $b_{6}$ are related to the transition temperature, while the $b_{7}$, $b_{8}$ and $b_{9}$ coefficients are related to the decrease in $V_{t}(T,p)$ and describe the shape of the crystalline transition. Specifically, $b_{5}$ represents the transition temperature at p=0, and $b_{6}$ represents the change in $T_{t}$ with pressure, and parameters $b_{7}$ and $b_{9}$ denote the radius of curvature and the change of the curvature radius with a pressure below $T_{t}$, respectively. Parameters $b_{1s}$ through $b_{4s}$ have the same meanings, but for the solid state.

In this study, the Tait parameters were determined by fitting the experimental *pVT* data to Equations (6)–(10) using the WinRheo software. The results are presented in Supplementary Table S7 with respect to the MWCNT loading. The specific volume at atmospheric pressure was obtained by extrapolating the data at pressures ranging from 10 to 1500 bar.

Figure 11 shows that the predicted specific volume (solid line) agrees very well with the experimental data (symbols), and the average relative error between the experimental and prediction values was well below 1%.

Within the investigated *p-T* experimental window, the transition temperature at atmospheric pressure is independent of MWCNT loading, i.e., the average value for $b_{5}$ is 102.5 °C (Supplementary Table S7). Therefore, it can be concluded that the onset of crystallization temperature from the DSC curves is consistent with the *pVT* onset of transition temperature at atmospheric pressure.

### 3.10. Density of LDPE/MWCNT Composite

Table 4 summarizes the density of the LDPE/MWCNT composite as a function of pressure, temperature and carbon nanotube loading, obtained from the *pVT* data (extreme conditions). The solid (bulk) density of these composites is also given in Table 4. It was found that the density of the LDPE/MWCNT composite increases with increasing pressure and decreases with increasing temperature. However, the effect of MWCNT loading does not seem to play a significant role up to 3 wt.%, as shown in Table 4.

The bulk density of the LDPE/MWCNT composite increased from 0.892 ± 0.0054 g/cm^3^ to 0.916 ± 0.0035 g/cm^3^ with increasing nanotube loading from 0.1 to 5 wt.% of MWCNT. In fact, a plateau at intermediate loading of 0.3–1 wt.% was observed. The difference in the bulk density of the LDPE/MWCNT composites might be due to the difference in crystallinity (the crystallinity ranges from 27.25% for 0.1 wt.% to 31.34% for 5 wt.%, as shown in Table 1), which results in a difference in the free volume contained within the amorphous phase [51]. The density of the LDPE polymer matrix was reported to be 0.913 g/cm^3^ [66].

### 3.11. Thermal Conductivity

**Figure 13:**
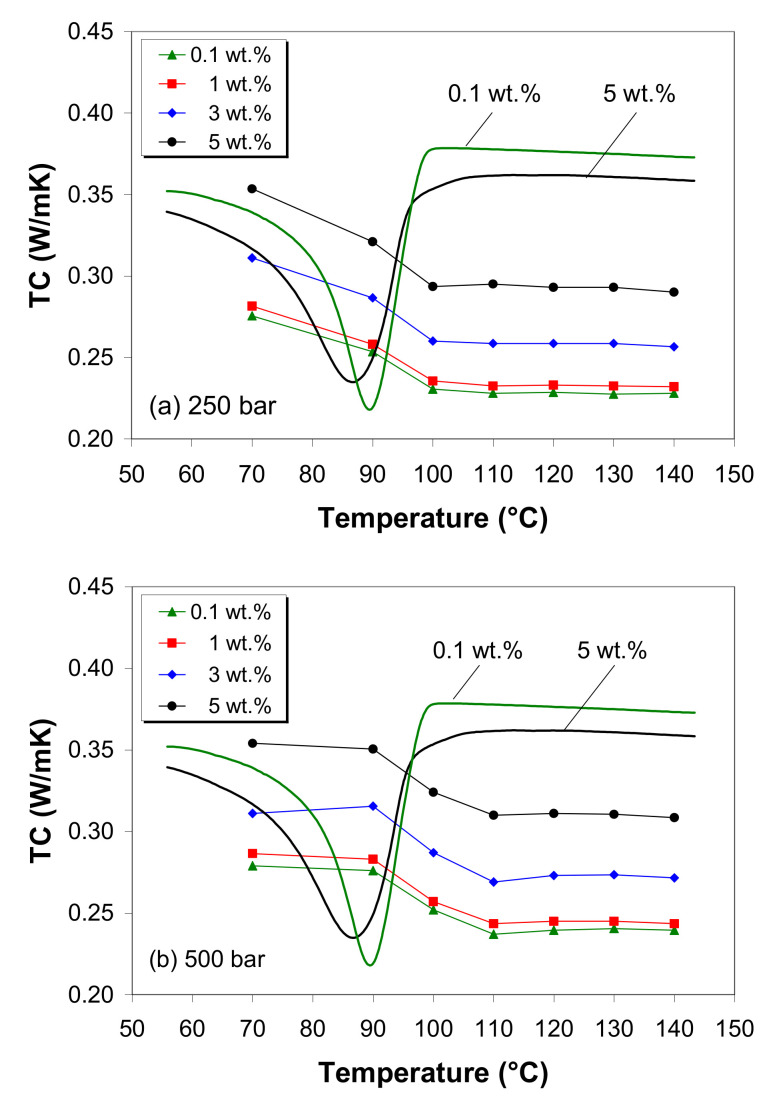
Thermal conductivity of the LDPE/MWCNT composites as a function of temperature and MWCNT wt.% at (**a**) 250 bar and (**b**) 500 bar.

Figure 13 shows the variation of the thermal conductivity of the LDPE/MWCNT composite as a function of MWCNT loading and the temperature measured at 250 and 500 bar. The test starts from completely melted samples, which crystallize during the isobaric measurements. Therefore, the DSC cooling curves for the lowest and highest carbon nanotube loadings are also shown in Figure 13. The thermal conductivity of the LDPE/MWCNT composite is influenced by the temperature and pressure, in addition to the nanotube loading, as shown in Figure 13, and reflects the morphological changes during the cooling of the composite melts.

During cooling under 250 bar, the thermal conductivity curves show two distinct characteristic regions (Figure 13a). In the melt state, assuming that the carbon nanotubes are separated by the polymer chains, the thermal conductivity is nearly independent of temperature up to the onset of the crystallization (e.g., 100 °C). Below this temperature, the thermal conductivity gradually increases with decreasing temperature up to the complete crystallization of the melts (e.g., 70 °C). The increase in thermal conductivity is attributed to the formation of crystals that enhance the heat transport mechanism [8,59].

On the other hand, during cooling under 500 bar, a three stage variation of thermal conductivity with temperature is observed, as illustrated in Figure 13b, indicating the impact of pressure on the crystallization behavior, as discussed in Section 3.8. In the temperature range of 110–140 °C, the temperature negligibly affects the thermal conducting behavior, whereas the thermal conductivity increases linearly with decreasing temperature until reaches its maximum value close to 90 °C, and then the curve tends to level off with a further decrease in temperature (i.e., plateau appearance) up to 70 °C.

When the effects of pressure and temperature were separated from that of the carbon nanotube, the results indicated a moderate enhancement in the thermal conductivity of the LDPE/MWCNT composite (25%–30%) with increasing MWCNT loading from 0.1 to 5 wt.% (Supplementary Table S8), although MWCNTs can exhibit thermal conductivity as high as 3000 W/m·K [28,29].

The increase in thermal conductivity with increasing MWCNT loading is mostly due to the fact that as the MWCNT loading increases, a denser nanotube network is formed, allowing phonon transport [7,17,27,60], and in part due to the effect of carbon nanotubes on the crystallization of the LDPE. In the solid state, the thermal conductivity of semi-crystalline polymers is affected by the crystallinity—due to the phonon scattering at the interface between the amorphous and crystalline phases [4,6,31], which in turn is affected by the CNT loading that provides nucleation sites for polymer crystallization and accelerates the crystal growth rate, as well as modifying the crystal size [2,10,12,20,23,55,68].

**Figure 14:**
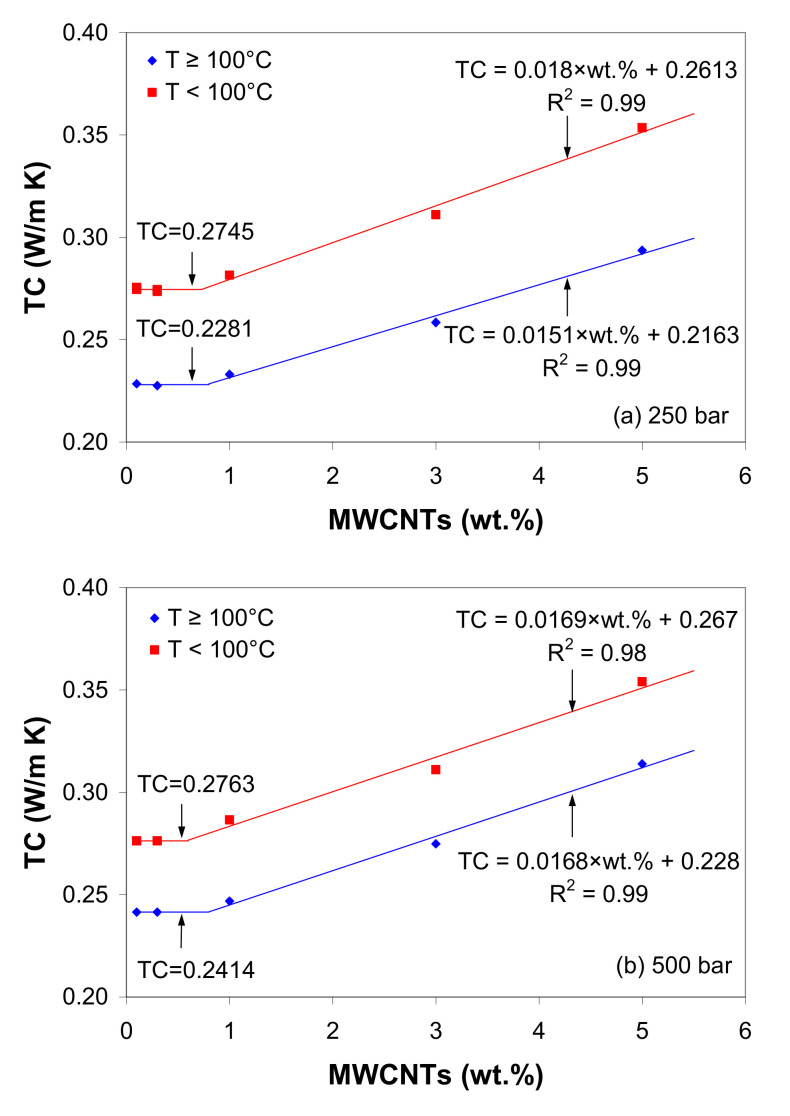
Effect of MWCNT loading on thermal conductivity of the LDPE/MWCNT composite at (**a**) 250 bar and (**b**) 500 bar. The solid lines show data fitting to linear models, whose parameters are given in the figure.

Figure 14a–b show the effect of nanotube loading on the thermal conductivity of the LDPE/MWCNT composite for 250 bar and 500 bar, respectively. The variation in thermal conductivity suggests two distinct zones, which hold for both the solid and melt states.

Below 1 wt.% of MWCNTs, the thermal conductivity of the LDPE/MWCNT composite is nearly constant with nanotube loading. In this region, carbon nanotubes do not really touch each other, and, since CNTs act as nuclei for crystallization, an insulating trans-crystalline layer around CNTs may form [75]. Therefore, the interface thermal resistance between the CNTs and LDPE matrix is larger than the critical value [8,31,59], and there are too few nanotubes for photons to move effectively. When the CNT loading is higher than 1 wt.%, the thermal conductivity is a monotonically increasing function of nanotube loading (Figure 14). The increase in thermal conductivity could be mostly related to the formation of a heat transport pathway between CNTs, which significantly facilitate the photons’ movement through the composites. Based on these considerations, it was concluded that 1 wt.% loading is in the region of the thermal percolation.

Regarding the effect of pressure, the experimental results indicate that the thermal conductivity increases with increasing pressure due to the fact that the pressure significantly reduces the inter-tube distance, reducing the contact between adjacent nanotubes, especially in the melt state, enhancing the photon transport.

## 4. Conclusions

In this work, the melt flow behavior of the LDPE/MWCNT composite (0.1 to 5 wt.%), including the specific volume and thermal conductivity, was investigated using capillary rheometry for conditions as experienced during melt processing. The goal was to provide relevant data (rheology models, i.e., the Cross and Carreau–Winter models; EoS, i.e., the Tait equation; and thermal conductivity) that might be used in CAE software. Based on the experimental results and the analytical models, the main findings can be summarized as follows:

- The LDPE/MWCNT composite features a strong increasing melt shear viscosity with increasing MWCNT loading beyond 1 wt.%, and this effect decreases with increasing shear rates due to solid-like behavior. Moreover, a sharp decrease in the Arrhenius flow activation energy is observed at MWCNT loading higher than 3 wt.%, reflecting the strong nanotube–nanoube interactions.
- The specific volume of the LDPE/MWCNT composites decreases with increasing MWCNT loading, suggesting an improvement of the shrinkage and warpage behavior in the presence of nanotubes.
- The thermal conductivity of the LDPE/MWCNT composite is nearly independent of nanotube loading up to the thermal percolation threshold of 1 wt.% and shows a linear increasing trend with further increases in nanotube loading.
- The Carreau–Winter and Cross viscosity models and the Tait equation capture very well the experimentally measured melt shear viscosity and the specific volume, respectively.

Despite some limitations (e.g., the effects of pressure on the melt shear viscosity and the effect of cooling rates on the specific volume were not investigated thoroughly, and the investigation was limited to the PLASTICYL^TM^ LDPE2001 masterbatch), the integration of these data in CAE applications could assist the polymer processing industry to improve polymer/CNT product and process development, especially injection molding.

## Figures and Tables

**Table 1:** The DSC parameters for the LDPE/MWCNT composites.

MWCNTs (wt.%)	1st Heating Scan			Cooling Scan			2nd Heating Scan		
	$T_{m}$ (°C)	$\Delta H_{m}$ (J/g)	χ (%)	$T_{c}$ (°C)	$\Delta H_{c}$ (J/g)	$T_{s}$ (°C)	$T_{m}$ (°C)	$\Delta H_{m}$ (J/g)	χ (%)
0.1	104.8	66.3	22.6	89.7	87.0	76.54	102.8	78.9	27.0
0.3	104.7	71.6	24.5	89.9	83.2	74.41	102.0	82.4	28.2
0.5	103.8	84.7	29.1	89.7	91.7	76.45	101.8	88.8	30.5
1	105.4	68.8	23.7	89.2	82.2	76.35	102.4	80.3	27.7
3	106.9	60.1	21.1	88.0	67.7	73.65	102.4	67.7	23.8
5	104.4	72.7	26.1	86.7	85.2	67.24	103.3	86.3	31.0

**Table 2:** Carreau–Winter parameters for master curves at a reference temperature of 130 °C.

Parameters	MWCNTs (wt.%)					
	0.1	0.3	0.5	1	3	5
$\eta_{0}$ (Pa·s)	211.04	276.09	271.01	259.91	280.30	653.36
λ (s)	0.008	0.013	0.012	0.009	0.007	0.020
$m_{c}$	0.565	0.550	0.552	0.574	0.619	0.626
$R^{2}$	0.999	0.999	0.999	0.999	0.988	0.999

**Table 3:** Cross parameters for master curves at a reference temperature of 130 °C.

Parameters	MWCNTs (wt.%)					
	0.1	0.3	0.5	1	3	5
$\eta_{0}$ (Pa·s)	276.55	381.76	380.72	332.03	395.09	1093.52
τ* (Pa)	38,364.58	28,160.93	28,595.41	40,465.30	42,938.44	29,112.84
λ (s)	0.01	0.01	0.01	0.01	0.01	0.04
n	0.379	0.398	0.400	0.362	0.347	0.354
$R^{2}$	1.00	1.00	0.999	0.997	0.989	0.999

**Table 4:** Density of the LDPE/MWCNT composites as a function of temperature and pressure.

MWCNTs (wt.%)	Temperature (°C)	*pVT* Density (g/cm^3^)		Bulk Density (kg/m^3^)
		10 bar	1500 bar	
0.1	30	0.951	0.997	0.892 ± 0.0054
	160	0.790	0.883	
0.3	30	0.954	1.000	0.895 ± 0.0033
	160	0.790	0.884	
0.5	30	0.938	0.984	0.896 ± 0.0046
	160	0.784	0.880	
1	30	0.950	0.996	0.896 ± 0.0043
	160	0.798	0.889	
3	30	0.947	0.993	0.905 ± 0.0075
	160	0.802	0.893	
5	30	0.960	1.005	0.916 ± 0.0035
	160	0.814	0.906	

## Supplementary Materials

The following are available online at https://www.mdpi.com/2073-4360/12/6/1230/s1, Table S1: Parameters of the power-law model, Table S2: Analysis of Variance for the consistency index *K*, Table S3: Analysis of Variance for power-law index *n*, Table S4: Flow activation energy, Table S5: Analysis of Variance for flow activation energy (kJ/mol), Table S6: Parameters of linear model *η = α + β · ϕ* and the goodness of fit, Table S7: Parameters of Tait equation for LDPE/MWCNT composites, Table S8: Thermal conductivity (W·m^−1^·K^−1^) of LDPE/MWCNT composites.
