# Supplementary material for: Experimental Investigation of the Melt Shear Viscosity, Specific Volume and Thermal Conductivity of Low-Density Polyethylene/Multi-Walled Carbon Nanotube Composites Using Capillary Flow
Source: Polymers (Basel). 2020 May 28;12(6):1230. doi: 10.3390/polym12061230 (PMC7361681; doi:10.3390/polym12061230)
Supplement: Supplementary file 1 [file polymers-12-01230-s001.pdf]

polymers

Supplementary Materials

Experimental Investigation of the Melt Shear Viscosity, Specific Volume and Thermal Conductivity of Low-Density Polyethylene/Multi-Walled Carbon Nanotube Composites Using Capillary Flow

Nicoleta-Violeta Stanciu, Felicia Stan * and Catalin Fetecau

Center of Excellence Polymer Processing, Dunarea de Jos University of Galati, 47 Domneasca, 800 008, Galati, Romania;

* Correspondence: felicia.stan@ugal.ro

Received: date; Accepted: date; Published: date

Table S1. Parameters of the power-law model.

MWCNTs (wt.%)	Temperature (°C)	K (Pa·s^n)	n	R^2
0.1	110	2392.76	0.549	0.994
	120	1523.35	0.585	0.996
	130	1021.41	0.617	0.995
	140	661.45	0.653	0.994
0.3	110	2703.96	0.534	0.996
	120	1749.85	0.570	0.996
	130	1132.40	0.605	0.996
	140	704.69	0.646	0.994
0.5	110	2870.78	0.527	0.995
	120	1845.44	0.563	0.996
	130	1226.03	0.596	0.996
	140	768.42	0.636	0.994
1	110	3553.04	0.504	0.995
	120	2519.42	0.526	0.998
	130	1674.94	0.560	0.996
	140	1125.64	0.591	0.996
3	110	4806.18	0.482	0.992
	120	3479.37	0.502	0.996
	130	2502.07	0.525	0.996
	140	1786.08	0.549	0.996
5	110	9086.57	0.419	0.996
	120	6327.03	0.446	0.997
	130	4721.72	0.467	0.998
	140	3669.44	0.483	0.999

Table S2. Analysis of variance for the consistency index *K*.

Source	DF	Seq SS	Adj SS	Adj MS	F	P	C (%)
MWCNT (wt.%)	5	59984628	59984628	11996926	33.66	0.000	65
Temperature (°C)	3	26266799	26266799	8755600	24.57	0.000	29
Residual Error	15	5346112	5346112	356407			
Total	23	91597539					

Table S3. Analysis of variance for the power-law index *n*.

Source	DF	Seq SS	Adj SS	Adj MS	F	P	C (%)
MWCNT (wt.%)	5	0.062143	0.062143	0.012429	145.86	0.000	69
Temperature (°C)	3	0.027216	0.027216	0.009072	106.47	0.000	30
Residual Error	15	0.001278	0.001278	0.000085			
Total	23	0.090637					

Table S4. Flow activation energy.

MWCNTs (wt.%)	App. shear rate (s^{-1})	$E_{\dot{\gamma}}$ (kJ/mol)	R^2
0.1	500	23.16	0.999
	1000	21.51	0.994
	3000	15.30	0.999
	4000	13.49	0.995
0.3	500	22.42	0.999
	1000	20.08	0.999
	3000	15.92	1.00
	4000	14.26	0.998
0.5	500	22.73	0.999
	1000	20.10	0.999
	3000	15.65	0.993
	4000	14.32	0.974
1	500	23.40	0.973
	1000	20.66	0.995
	3000	15.03	0.963
	4000	13.64	0.935
3	500	22.03	0.984
	1000	20.77	0.990
	3000	15.36	0.943
	4000	13.91	0.908
5	500	19.10	0.992
	1000	15.72	0.999
	3000	9.21	0.953
	4000	7.83	0.920

Table S5. Analysis of variance for the flow activation energy (kJ/mol).

Source	DF	Seq SS	Adj SS	Adj MS	F	P	%
MWCNTs (wt.%)	5	91.141	91.141	18.228	39.01	0.000	21
App. shear rate (1/s)	3	344.022	344.022	114.674	245.41	0.000	78
Residual Error	15	7.009	7.009	0.467			
Total	23	442.173					

Table S6. Parameters of the linear model $\eta = \alpha + \beta \cdot \phi$ and the goodness of fit.

Melt temperature (°C)	App. shear rate (s^{-1})	α (Pa·s)	β (Pa·s/wt.%)	R^2
110	250	205.25	32.11	0.984
	500	151.36	19.68	0.984
	1000	114.11	11.54	0.986
	3000	63.51	4.52	0.996
	4000	52.65	3.64	0.991
120	250	205.25	32.11	0.983
	500	122.53	15.97	0.975
	1000	91.59	10.00	0.987
	3000	53.64	4.14	0.993
	4000	45.62	3.24	0.998
130	250	130.31	23.49	0.985
	500	100.38	14.60	0.974
	1000	73.75	12.56	0.983
	3000	45.91	3.87	0.986
	4000	39.29	3.11	0.981
140	250	102.84	20.93	0.963
	500	81.69	12.89	0.984
	1000	63.56	8.17	0.984
	3000	37.59	4.13	0.983
	4000	33.91	2.94	0.974

[1] η - apparent melt shear viscosity, ϕ - MWCNT wt.%, α, β - model parameters.

Table S7. Parameters of the Tait model for the LDPE/MWCNT composites.

Parameters	MWCNTs (wt.%)					
	**0.1**	**0.3**	**0.5**	**1**	**3**	**5**
b_{1s} (mm^3/g)	1.1018×10^{3}	1.0968×10^{3}	1.0975×10^{3}	1.0940×10^{3}	1.0815×10^{3}	1.0060×10^{3}
b_{2s} (mm^3/kg °C)	7.8597×10^{-1}	7.6150×10^{-1}	6.2898×10^{-1}	7.1563×10^{-1}	5.3723×10^{-1}	1.0166×10^{-1}
b_{3s} (bar)	1.0294×10^{3}	1.0945×10^{3}	1.0742×10^{3}	1.1483×10^{3}	1.1377×10^{3}	1.8648×10^{3}
b_{4s} (1/°C)	1.1966×10^{-2}	1.0896×10^{-2}	1.2118×10^{-2}	1.0081×10^{-2}	1.1757×10^{-2}	1.0488×10^{-2}
b_{1m} (mm^3/g)	1.1863×10^{3}	1.1782×10^{3}	1.2029×10^{3}	1.1844×10^{3}	1.1900×10^{3}	1.1739×10^{3}
b_{2m} (mm^3/kg °C)	1.2794	1.3516	1.1170	1.2138	1.0137	9.3332×10^{-1}
b_{3m} (bar)	7.1798×10^{2}	7.2243×10^{2}	7.5580×10^{2}	8.3479×10^{2}	8.6149×10^{2}	8.5314×10^{2}
b_{4m} (1/°C)	5.0292×10^{-4}	4.8950×10^{-4}	1.6284×10^{-3}	3.5456×10^{-5}	3.7832×10^{-4}	3.1927×10^{-3}
b_5 (°C)	102.51	101.55	102.40	102.51	102.83	102.72
b_6 (°C/bar)	2.0395×10^{-2}	2.1096×10^{-2}	2.0329×10^{-2}	2.2039×10^{-2}	1.9518×10^{-2}	1.9167×10^{-2}
b_7 (mm^3/g)	7.1139×10^{1}	6.6160×10^{1}	9.0432×10^{1}	7.3962×10^{1}	9.2788×10^{1}	1.5111×10^{2}
b_8 (1/°C)	3.3673×10^{-2}	3.5145×10^{-2}	2.6435×10^{-2}	2.6435×10^{-2}	2.6558×10^{-2}	1.6866×10^{-2}
b_9 (1/bar)	5.6669×10^{-4}	5.8702×10^{-4}	4.4469×10^{-4}	4.7290×10^{-4}	4.4272×10^{-4}	3.8001×10^{-4}

Table S8. Thermal conductivity (W/m·K) of the LDPE/MWCNT composites.

Pressure (bar)	Temperature (°C)	MWCNTs (wt.%)				
		**0.1**	**0.3**	**1**	**3**	**5**
100	70	0.272	0.272	0.280	0.312	0.343
	90	0.240	0.238	0.243	0.269	0.300
	110-140	0.223	0.222	0.226	0.251	0.282
250	70	0.276	0.274	0.282	0.311	0.354
	90	0.254	0.252	0.258	0.287	0.321
	110-140	0.229	0.228	0.233	0.258	0.293
500	70	0.279	0.274	0.287	0.311	0.354
	90	0.276	0.275	0.283	0.316	0.351
	100	0.252	0.252	0.257	0.287	0.324
	110-140	0.239	0.238	0.244	0.272	0.310
